# Multicellular cooperation and the hallmarks of cancer: A new foundation

**DOI:** 10.1093/emph/eoaf026

**Published:** 2025-09-19

**Authors:** Carlo C Maley, Amy M Boddy, Aurora M Nedelcu, Athena Aktipis

**Affiliations:** Arizona Cancer Evolution Center, Biodesign Institute Arizona State University, Tempe, AZ 85281, USA; Center for Evolution and Medicine, Arizona State University, Tempe, AZ 85281, USA; Biodesign Center for Biocomputing, Security and Society, Arizona State University, Tempe, AZ, 85281, USA; School of Life Sciences, Arizona State University, Tempe, AZ 85281, USA; Arizona Cancer Evolution Center, Biodesign Institute Arizona State University, Tempe, AZ 85281, USA; Department of Anthropology, University of California Santa Barbara, Santa Barbara, CA, 93106-3210, USA; Department of Biology, University of New Brunswick, Fredericton, NB E3B 5A3, Canada; Arizona Cancer Evolution Center, Biodesign Institute Arizona State University, Tempe, AZ 85281, USA; Center for Evolution and Medicine, Arizona State University, Tempe, AZ 85281, USA; Department of Psychology, Arizona State University, Tempe, AZ, 85281, USA; Global Futures Lab, Arizona State University, Tempe, AZ, 85281, USA

**Keywords:** cooperation, hallmarks, cancer, mulitcellularity, evolution

## Abstract

Multicellularity evolved independently several times across the tree of life. In all cases, these events were dependent on various types of cellular cooperation. We previously identified five universal foundations of cellular cooperation that are required in all complex multicellular lineages to allow the collection of cells to function and reproduce as a whole (i.e. a multicellular individual). These include: proliferation inhibition, controlled cell death, resource allocation, division of labor, and maintenance of the extracellular environment. We propose that there is a sixth universal foundation of multicellularity that breaks down in cancer: proximity maintenance. By staying in close proximity, cells can more easily provide benefits for one another, communicate, coordinate their behavior, and evolve increased size and complexity. Here, we revisit and further develop the five original foundations of multicellular cooperation in the context of the evolution of multicellularity from unicellular ancestors and their implications for cancer progression. In our previous work, we suggested that the breakdown of all these cooperative behaviors is reflected in the universal hallmarks of cancer. Similarly, the breakdown of proximity maintenance maps to another hallmark of cancer—activating invasion and metastasis.

## INTRODUCTION

In 2015, we proposed that cancer could be considered as a breakdown of five foundations of multicellular cooperation [1]. These five foundations of multicellular cooperation are proliferation inhibition, controlled cell death, resource allocation, division of labor, and maintenance of the extracellular environment. In that 2015 paper, we showed how the breakdown of each of these foundations corresponds to universal characteristics of cancer, as described by Hanahan and Weinberg in their classic papers on the hallmarks of cancer [2]. Here, we review the five original foundations of multicellular cooperation. We propose a subtle but important revision to this framework: acknowledging that the foundations of multicellular cooperation were likely co-opted from unicellular behaviors that became regulated in ways that were beneficial for the multicellular collective. Then, we suggest that there is likely a sixth foundation of multicellular cooperation: proximity maintenance; and that the breakdown of proximity maintenance contributes to the cancer phenotypes of invasion and metastasis.

## REVISITING THE ORIGINAL FIVE FOUNDATIONS OF MULTICELLULAR COOPERATION AND THE CO-OPTION OF UNICELLULAR BEHAVIORS

Across all forms of multicellularity, cancer or cancer-like phenomena can result from the breakdown of cellular cooperation. Complex obligate multicellularity (defined by the stable presence of specialized cell types) evolved independently at least ten times: once in animals, at least twice in green plants, twice in red algae, twice in brown algae, and at least three (but as many as 8–11) times in fungi [3, 4]. We found cancer and/or cancer-like phenomena ubiquitous across all independently evolved multicellular lineages [1].

Each of the five foundations of multicellular cooperation that we identified in our 2015 paper likely were co-opted from unicellular behaviors that were beneficial for unicellular organisms in certain circumstances. With the emergence of multicellularity, what evolved was the capacity to regulate these cooperative behaviors in ways that benefited the multicellular collective. Versions (at a mechanistic level) of most of the previously identified foundations can be found in unicellular organisms (see Fig. 1 in ref [5]).

**Figure 1:**
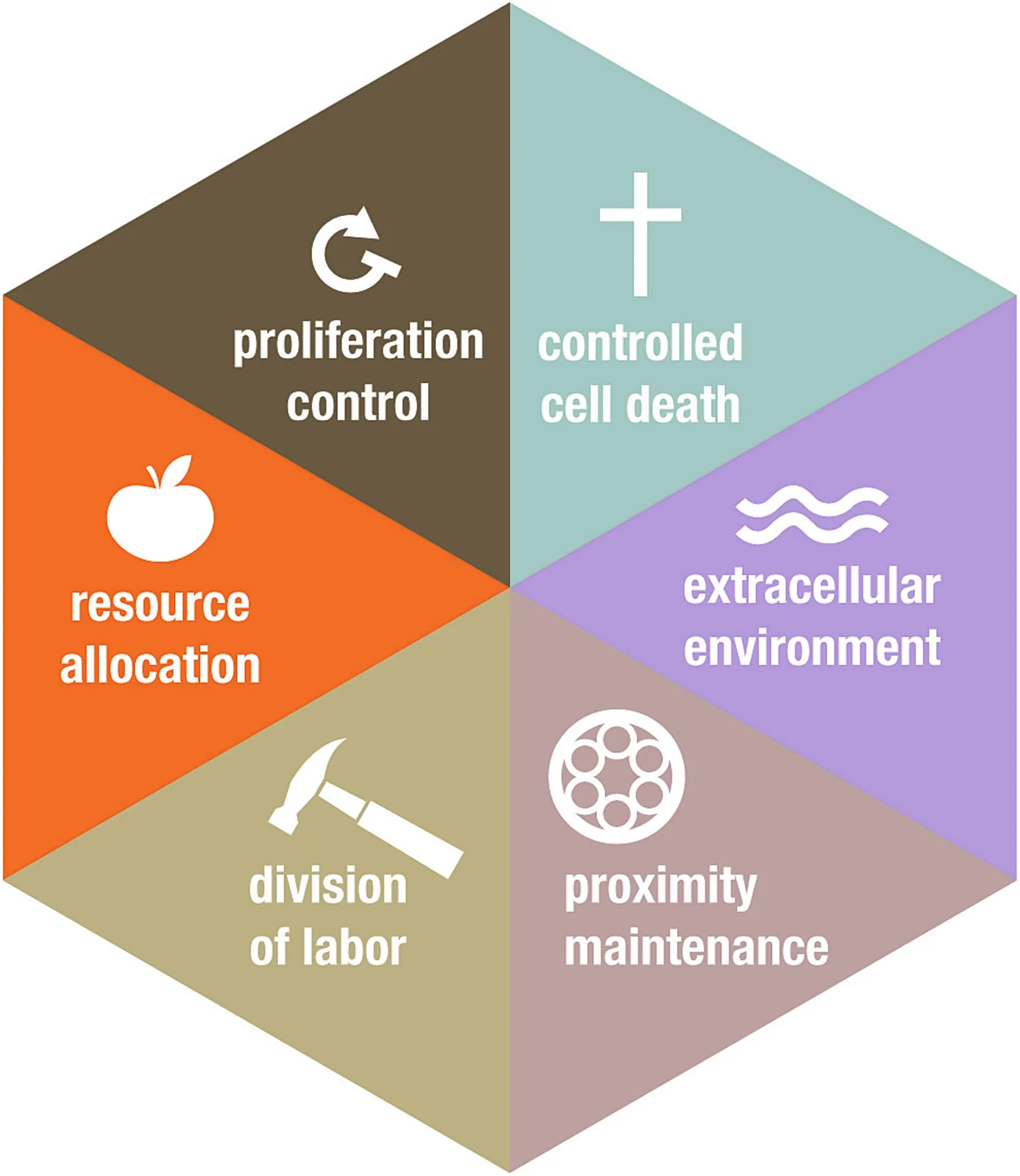
The six proposed foundations of multicellular cooperation are all forms of cellular cooperation that are necessary for the evolution and maintenance of multicellularity. Each time complex multicellularity has evolved in the history of life, it has been built upon these six foundations. The extracellular environment in a multicellular organism includes all the components of the tissue microenvironment as well as the systemic factors involved in maintaining that environment.

*Proliferation control:* The capacity for inhibiting proliferation allows for the control and regulation of cellular division that enables the building and maintaining of a complex multicellular body. However, it is not just the inhibition of proliferation but the *regulation* of cell proliferation in response to developmental and homeostatic signals that is necessary for building and maintaining a multicellular body. This capacity for regulation of cell proliferation exists in unicellular organisms as well. They evolved a variety of mechanisms to adaptively regulate their proliferation in response to changes in their environment or via cues from other members of the population (i.e. quorum sensing; see discussion in [6]). These mechanisms may have been co-opted by early multicellular organisms to regulate cell proliferation for the benefit of the group of cells and in response to cues from the group [5, 7]. Thus, an innovation in the evolution of multicellularity was the ability to regulate (and even inhibit—as in terminally differentiated cells) cell proliferation in response to group signals (in addition to environmental signals) in a way that benefits the group, even when those responses are costly to individual cells (e.g. altruistic responses).

*Controlled cell death*: Complex multicellular organisms generally use controlled cell death in the development and maintenance of the body. There is evidence of controlled cell death (often also referred to as programmed cell death or PCD) in a variety of unicellular organisms, which appears to be an ancient phenomenon [8–12]. Again, the novelty in multicellular organisms lies in the ability to control cell death in response to group cues and for the benefit of the group; for example, during development and when mutated cells undergo apoptosis so that they do not increase the cancer risk to the organism.

*Maintenance of the extracellular environment*: Multicellular organisms are more than just a collection of cells. They include extracellular structures such as basal membranes, extracellular matrix, chitin, cellulose, cartilage, and bone, not to mention chemical signals. Proper functioning of a multicellular organism requires production and maintenance of that extracellular environment, as well as removal of waste products. Bacterial biofilms exhibit many of these features, including coordination through chemical signaling and production of complex extracellular matrices [13].

*Resource allocation*: One common feature of multicellular organisms is that many of the cells in the multicellular body do not have direct access to resources. This has led to the evolution of a variety of structures for delivering resources throughout the body [14]. Microbial biofilms also feature extracellular structures and channels for resource delivery [15, 16].

*Division of labor*: Part of the definition of complex multicellularity is the differentiation of cells into different cell types specialized for different functions. This appears to be one of the primary advantages of multicellularity [17]. However, this too is not unique to multicellular organisms. Microbes in biofilms also differentiate, and even outside of biofilms may differentiate in response to environmental signals or progression through a life cycle [13, 18–20].

These foundations of multicellularity were likely co-opted during the evolution of multicellularity from adaptations at the unicellular level where cooperation provided a selective advantage in specific contexts (e.g. environmental stress, resource limitation). However, these traits can manifest differently when expressed at the level of the multicellular organism because they are often stably expressed at the multicellular level as part of multicellular development. In other words, they are genuine, heritable, group-level adaptations.

In the context of cancer, mutations in all these cooperative mechanisms can increase cell-level fitness (i.e. selfish behavior of potentially cancerous cells) while decreasing the fitness of the multicellular organism. However, because many of the genes used in cellular cooperation evolved from genes involved in processes that increase cell-level fitness in unicellular life, mutations in multicellular cooperative mechanisms might also decrease cell-level fitness. In fact, such cell-level costs are thought to have contributed to the stabilization of cooperative behaviors during the evolution of multicellularity, by decreasing the fitness of cheaters [21, 22]. Mutations in pathways co-opted from processes increasing the fitness of unicellular ancestors could prevent cancer cells from responding appropriately to their environment. This might be exploited to decrease the fitness of cancer cells for clinical benefit [5, 6].

For instance, in contrast to healthy cells, cancer cells often fail to, or only partially respond to limiting conditions (e.g. short-term starvation, low glucose/low serum) and continue to proliferate rather than halting their cell cycles to prevent damage from those stresses. Fasting can exploit this difference, shutting down the proliferation of normal cells but not cancer cells, thereby protecting normal cells and sensitizing cancer cells to chemotherapies that target proliferating cells [23]. Furthermore, fasting cycles can not only improve the efficiency and safety of chemotherapy, but also retard tumor growth [24].

## A SIXTH FOUNDATION OF MULTICELLULAR COOPERATION: PROXIMITY MAINTENANCE

In addition to our previously identified foundations of multicellular cooperation, we are proposing another foundation (perhaps the most basic foundation since it also applies to simple multicellularity): cells must maintain their *proximity* in order to benefit from their cooperative activities and function as a coherent entity (Fig. 1) [19]. Cell-to-cell adhesion has long been recognized as fundamental to the evolution of multicellularity [25, 26]. Typically, this has been achieved by cells staying together after cell division, and often by constructing a boundary between them and the rest of the environment in the form of skin/epidermis, cuticle, or some other boundary structure.

Similar to the other foundations of multicellular cooperation, unicellular organisms also have mechanisms to maintain proximity, particularly in the form of biofilms. They maintain proximity through the construction of an extracellular matrix (ECM) and/or adhesion molecules [27, 28]. Many components of ECM and adhesion molecules are also found in unicellular organisms. For instance, choanoflagellates (which include the closest unicellular relatives on animals) express some of the important adhesion molecules in animals (e.g. cadherins), whose function appears to be in sensing and responding to extracellular cues [26].

Maintaining proximity allows cells to cooperate and function as a coherent entity [19]. Remaining in close proximity following cell division also allows for positive assortment (cooperators interact with one another more often than they would at random) and kin selection, which are general principles that allow for the evolution of stable cooperation. This can lead to a positive feedback loop in which positive assortment selects for cooperation and cooperation selects for positive assortment [29]. Furthermore, proximity maintenance also facilitated the transition from a group of cells to a new unit of selection [30, 31].

In more complex multicellular systems, a variety of additional mechanisms for maintaining proximity in a dynamic context evolved to both exclude non-cooperators and increase functionality in the group. For instance: immune systems and complex molecular tagging systems to distinguish self from non-self (e.g. the major histocompatibility complex) [32, 33]; organization into tissues by expressing tissue-specific adherence molecules [3] (e.g. cadherins) and/or attaching to basal membranes [3] (i.e. epithelia); creating complex structures [3] (e.g. during development) or functional networks (e.g. neural networks) through responding to extracellular gradients or electrical signals [34].

However, enforcing proximity also leads to new challenges for multicellular cooperation because cells cannot “walk away” (i.e. leave cheaters behind) when cheating occurs [35]. Rather, multicellular organisms must have mechanisms for monitoring and responding to cheating *in situ*. This likely led to the evolution of complex systems to detect and remove cheating cells, including the immune system.

## METASTASIS AS A BREAKDOWN OF THE SIXTH FOUNDATION OF MULTICELLULAR COOPERATION

In our original conception, we mapped many of the hallmarks of cancer to the breakdown of five different forms of multicellular cooperation [1], showing that each foundation was associated with several known characteristics of cancer. This additional foundation of multicellular cooperation, proximity maintenance, allows us to map another hallmark—metastasis (i.e. cell detachment and migration from the original site), to the breakdown of another form of cellular cooperation.

The reason that the hallmarks of cancer are universal to all cancers, and keep evolving independently in each cancer, is that the breakdown of cooperation (i.e. cheating) provides a fitness advantage to the neoplastic cells (allowing selection to act at the cell level). In our previous analysis, we found a natural association of each hallmark with a form of cheating on each type of cellular cooperation that gives a neoplastic cell a fitness advantage, with the exception of invasion/migration and metastasis.

At the time, it wasn’t clear how invasion and metastasis could be a form of cheating on any of the original five foundations of multicellular cooperation. We associated it with cheating on the maintenance of the extracellular environment because invasion often involves disrupting the basement membrane (in animals). With the recognition of proximity maintenance as a missing foundation of multicellular cooperation, invasion and metastasis can be seen as cheating in this specific form of cooperation (Fig. 2). Invasive and metastatic cells are no longer staying with the cooperating cells of their tissue of origin.

**Figure 2:**
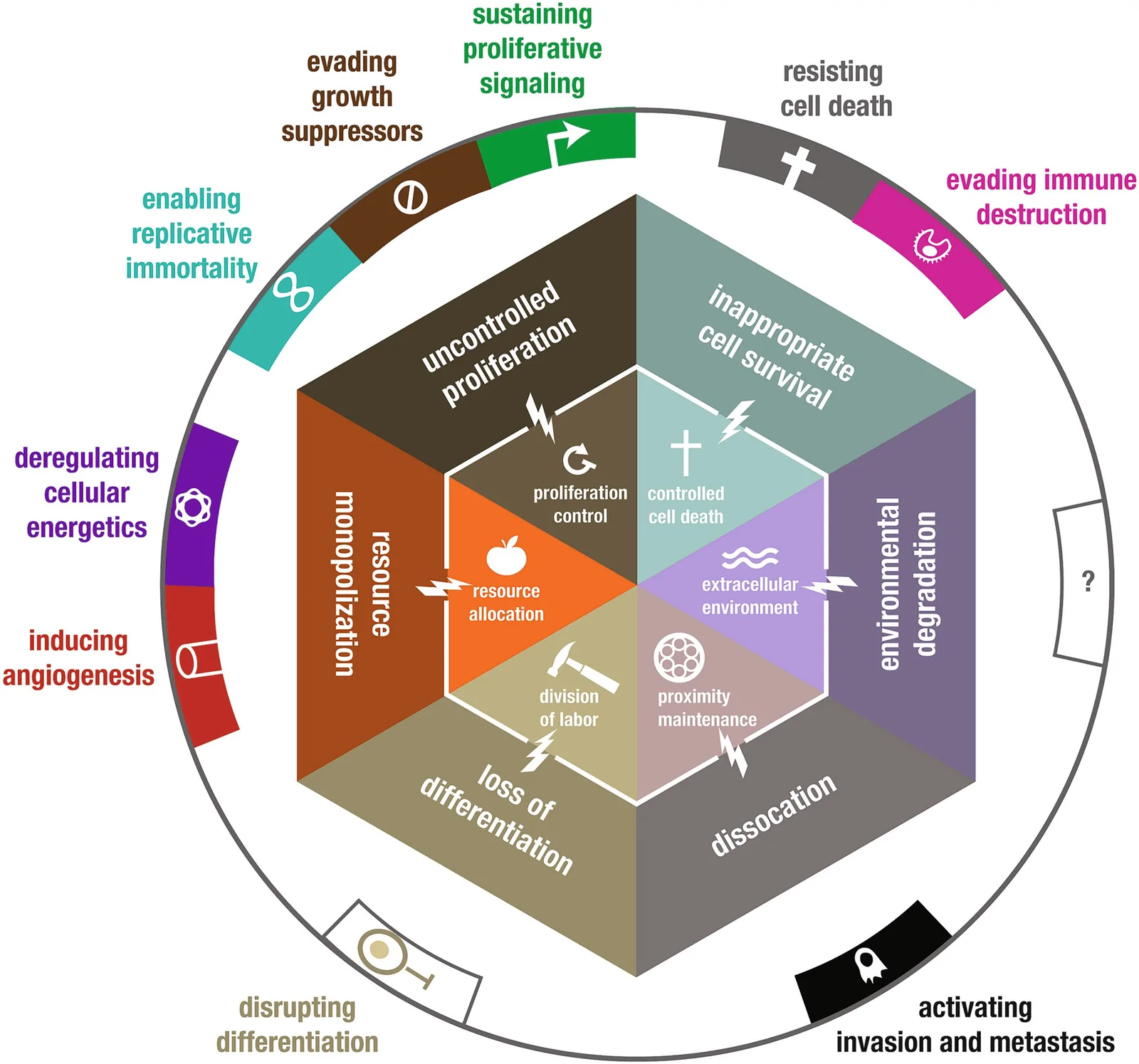
The six universal foundations of multicellular cooperation (inner hexagon) are all necessary for the development and maintenance of complex multicellular organisms. With cooperation comes the potential for cheating, and so each form of cooperation is paired with a form of cheating that is seen in cancers (outer hexagon). Because cheating generally provides a cell-level fitness advantage within the context of a multicellular organism, multiple forms of cellular cheating have been recognized as hallmarks of cancer [2]. This perspective suggests that we have missed at least one hallmark (disrupting differentiation) and perhaps another (loss of extracellular maintenance).

Invasion and metastasis might not just be about a breakdown of proximity maintenance within the multicellular body; it can also involve proximity maintenance of cancer cells with one another. Cancer cells can cooperate with one another to stay together during invasion and metastasis, which can make invasion and metastasis more successful and result in increased fitness for the cancer cells [36, 37]. For instance, tumor cell clusters, which are groups of cooperative cancer cells that detach and migrate as multicellular units, have been associated with increased metastatic potential [37–43].

Across other foundations of multicellular cooperation, we see this kind of pattern, with cancer cells evolving not just to cheat in the foundations of multicellular cooperation, but also to use these capacities for cooperation with other cancer cells [44]. For example, cancer cells appear to engage in division of labor and synergistic inter-clonal cooperation, which facilitates metastasis [45]. Thus, the re-evolution of cooperation among cancer cells is likely to be a major challenge for multicellular bodies, and therefore also for treating cancer, especially in its advanced stages [36]. This suggests that there may be clinical benefit in disrupting the ability of cancerous cells to maintain proximity with each other thus limiting their capacities to cooperate with one another. Those potential benefits would have to be weighed against the risk of facilitating metastasis through the dissemination of cancer cells.

## IDENTIFYING THE FOUNDATIONS OF MULTICELLULAR COOPERATION HELPS US DISCOVER HALLMARKS OF CANCER

One useful consequence of approaching the hallmarks of cancer from the first principles of the foundations of multicellular cooperation is that it suggests that we may be missing some hallmarks (Fig. 2). We have argued elsewhere that cheating on the division of labor, realized as the disruption of differentiation, is a missing hallmark [46]. This was recently supported by Hanahan [47] in what he called “unlocking phenotypic plasticity.” His description of this putative hallmark is based on three different versions of disruption of differentiation: dedifferentiation, blocked differentiation, and transdifferentiation [47]. But the mapping of hallmarks to the foundations of multicellularity in Fig. 2 also suggests that we may be missing an additional hallmark in the loss of the maintenance of the extracellular environment. Neoplastic cells could theoretically gain a fitness advantage by failing to contribute to the building of extracellular structures and instead devoting those resources to their own reproduction. Cancer cells may also gain an advantage from not contributing to homeostatic systems that maintain their microenvironment (e.g. pH, oxygen, other nutrients, and cytokines) and may even gain an advantage from disrupting those systems. For instance, acidification of the tumor microenvironment associated with the preferential use of glycolysis (resulting in the release of lactic acid) can induce cell detachment and metastasis [48, 49] and may contribute to increased cell proliferation [50]. Disruptions of some homeostatic systems might also feed into neoplastic cells’ monopolization of resources and uncontrolled proliferation. In animals, maintenance of extracellular structures is primarily handled by fibroblasts (for extracellular matrix), chondroblasts (for cartilage) and osteoblasts (for bone). Thus, fibroblasts, chondroblasts, and osteoblasts might gain a cellular fitness advantage by stopping their metabolic investments in the production of those structures. Such cheating would likely be selected for during neoplastic progression of the sarcomas that evolve from those cells. Investigating disruptions in the extracellular environment (as well as other forms of cellular cheating) as markers of potential precancerous lesions may provide new avenues of research and cancer prevention.

## Conclusion

Cancer is a disease of multicellularity. It is a form of breakdown in the principles underlying multicellularity itself. By investigating and understanding the mechanisms that establish and maintain multicellularity, such as regulation of cell proliferation and death, cell proximity, and maintaining an extracellular environment, we can gain novel perspectives on the fundamental nature of cancer. This view of cancer as a breakdown in multicellular cooperation allows us to highlight potential missing hallmarks of cancer, organize and think about mechanisms of cancer in a new light, and ultimately can reveal new avenues of research with cancer prevention and therapeutic implications.

## References

[1] Aktipis CA, Boddy AM, Jansen G et al. Cancer across the tree of life: Cooperation and cheating in multicellularity. *Philos Trans R Soc Lond Ser B Biol Sci* 2015;370:20140219. 10.1098/rstb.2014.0219 PMC4581024 26056363

[2] Hanahan D, Weinberg RA. Hallmarks of cancer: The next generation. *Cell.* 2011;144:646–74. 10.1016/j.cell.2011.02.013 21376230

[3] Niklas K, Newman S. The many roads to and from multicellularity. *J Exp Bot* 2019;71:3247–53. 10.1093/jxb/erz547 PMC7289717 31819969

[4] Nagy LG, Kovács GM, Krizsán K. Complex multicellularity in fungi: Evolutionary convergence, single origin, or both? *Biol Rev Camb Philos Soc* 2018;93:1778–94. 10.1111/brv.12418 29675836

[5] Nedelcu AM . The evolution of multicellularity and cancer: Views and paradigms. *Biochem Soc Trans* 2020;48:1505–18. 10.1042/BST20190992 32677677

[6] Nedelcu AM. Understanding Ancient Legacies to Expose and Exploit Cancer’s Evolutionary Vulnerabilities. In: Ujvari B, Roche B, Thomas F (eds.), Ecology and evolution of cancer 2017 (pp. 203–209). San Diego: Academic Press. https://www.sciencedirect.com/science/article/pii/B9780128043103000156

[7] Nedelcu AM, Michod RE. Stress responses co-opted for specialized cell types during the early evolution of multicellularity: The role of stress in the evolution of cell types can Be traced back to the early evolution of multicellularity. *Bioessays.* 2020;42:e2000029. 10.1002/bies.202000029 32163611

[8] Ramsdale M . Programmed cell death in the cellular differentiation of microbial eukaryotes. *Curr Opin Microbiol* 2012;15:646–52. 10.1016/j.mib.2012.09.005 23083808

[9] Debrabant A, Lee N, Bertholet S et al. Programmed cell death in trypanosomatids and other unicellular organisms. *Int J Parasitol* 2003;33:257–67. 10.1016/S0020-7519(03)00008-0 12670511

[10] Bidle KD . Programmed cell death in unicellular phytoplankton. *Curr Biol* 2016;26:R594–607. 10.1016/j.cub.2016.05.056 27404255

[11] Durand PM, Ramsey G. The concepts and origins of cell mortality. *Hist Philos Life Sci* 2023;45:23. 10.1007/s40656-023-00581-8 PMC10250477 37289372

[12] Durand PM, Ramsey G. The nature of programmed cell death. *Biol Theory* 2019;14:30–41. 10.1007/s13752-018-0311-0

[13] Brunet T, King N. The origin of animal multicellularity and cell differentiation. *Dev Cell* 2017;43:124–40. 10.1016/j.devcel.2017.09.016 PMC6089241 29065305

[14] Bikfalvi A . Evolution of the Vascular System. A Brief History of Blood and Lymphatic Vessels. Cham: Springer International Publishing, 2017. 35–41.

[15] Quan K, Hou J, Zhang Z et al. Water in bacterial biofilms: Pores and channels, storage and transport functions. *Crit Rev Microbiol* 2022;48:283–302. 10.1080/1040841X.2021.1962802 34411498

[16] Flemming H, Wingender J. The biofilm matrix. *Nat Rev Microbiol* 2010;8:623–33. 10.1038/nrmicro2415 20676145

[17] Niklas KJ . The evolutionary-developmental origins of multicellularity. *Am J Bot* 2014;101:6–25. 10.3732/ajb.1300314 24363320

[18] Webb JS, Givskov M, Kjelleberg S. Bacterial biofilms: Prokaryotic adventures in multicellularity. *Curr Opin Microbiol* 2003;6:578–85. 10.1016/j.mib.2003.10.014 14662353

[19] Knoll AH . The multiple origins of complex multicellularity. *Annu Rev Earth Planet Sci* 2011;39:217–39. 10.1146/annurev.earth.031208.100209

[20] Giri S, Waschina S, Kaleta C et al. Defining division of labor in microbial communities. *J Mol Biol* 2019;431:4712–31. 10.1016/j.jmb.2019.06.023 31260694

[21] Foster KR, Shaulsky G, Strassmann JE et al. Pleiotropy as a mechanism to stabilize cooperation. *Nature.* 2004;431:693–6. 10.1038/nature02894 15470429

[22] Cameron-Pack ME, König SG, Reyes-Guevara A et al. A personal cost of cheating can stabilize reproductive altruism during the early evolution of clonal multicellularity. *Biol Lett* 2022;18:20220059. 10.1098/rsbl.2022.0059 PMC9213111 35728616

[23] Raffaghello L, Lee C, Safdie FM et al. Starvation-dependent differential stress resistance protects normal but not cancer cells against high-dose chemotherapy. *Proc Natl Acad Sci USA* 2008;105:8215–20. 10.1073/pnas.0708100105 PMC2448817 18378900

[24] Lee C, Raffaghello L, Brandhorst S et al. Fasting cycles retard growth of tumors and sensitize a range of cancer cell types to chemotherapy. *Sci Transl Med* 2012;4:124ra27. 10.1126/scitranslmed.3003293 PMC3608686 22323820

[25] Niklas KJ, Newman SA. The origins of multicellular organisms. *Evol Dev* 2013;15:41–52. 10.1111/ede.12013 23331916

[26] Abedin M, King N. Diverse evolutionary paths to cell adhesion. *Trends Cell Biol* 2010;20:734–42. 10.1016/j.tcb.2010.08.002 PMC2991404 20817460

[27] Nwoko E-SQA, Okeke IN. Bacteria autoaggregation: How and why bacteria stick together. *Biochem Soc Trans* 2021;49:1147–57. 10.1042/BST20200718 PMC8286834 34110370

[28] Doloman A, Sousa DZ. Mechanisms of microbial co-aggregation in mixed anaerobic cultures. *Appl Microbiol Biotechnol* 2024;108:407. 10.1007/s00253-024-13246-8 PMC11224092 38963458

[29] Fletcher JA, Doebeli M. A simple and general explanation for the evolution of altruism. *Proc Biol Sci* 2009;276:13–9. 10.1098/rspb.2008.0829 PMC2614248 18765343

[30] Maynard Smith J, Szathmary E. The Major Transitions in Evolution. Oxford, UK: Oxford University Press, 1995.

[31] Clarke E . Levels of selection in biofilms: Multispecies biofilms are not evolutionary individuals. *Biol Philos* 2016;31:191–212. 10.1007/s10539-016-9517-3

[32] Tsutsui ND . Scents of self: The expression component of self/non-self recognition systems. *Ann Zool Fennici* 2004;41:713–27.

[33] De Tomaso AW, Nyholm SV, Palmeri KJ et al. Isolation and characterization of a protochordate histocompatibility locus. *Nature.* 2005;438:454–9. 10.1038/nature04150 PMC1401502 16306984

[34] Levin M . Bioelectric networks: The cognitive glue enabling evolutionary scaling from physiology to mind. *Anim Cogn* 2023;26:1865–91. 10.1007/s10071-023-01780-3 PMC10770221 37204591

[35] Aktipis CA . Is cooperation viable in mobile organisms? Simple walk away rule favors the evolution of cooperation in groups. *Evol Hum Behav* 2011;32:263–76. 10.1016/j.evolhumbehav.2011.01.002 PMC3110732 21666771

[36] Capp J-P, Thomas F, Marusyk A et al. The paradox of cooperation among selfish cancer cells. *Evol Appl* 2023;16:1239–56. 10.1111/eva.13571 PMC10363833 37492150

[37] Naffar-Abu Amara S, Kuiken HJ, Selfors LM et al. Transient commensal clonal interactions can drive tumor metastasis. *Nat Commun* 2020;11:5799. 10.1038/s41467-020-19584-1 PMC7669858 33199705

[38] Aceto N, Bardia A, Miyamoto DT et al. Circulating tumor cell clusters are oligoclonal precursors of breast cancer metastasis. *Cell.* 2014;158:1110–22. 10.1016/j.cell.2014.07.013 PMC4149753 25171411

[39] Cheung KJ, Padmanaban V, Silvestri V et al. Polyclonal breast cancer metastases arise from collective dissemination of keratin 14-expressing tumor cell clusters. *Proc Natl Acad Sci USA* 2016;113:E854–63. 10.1073/pnas.1508541113 PMC4763783 26831077

[40] Harryman WL, Hinton JP, Rubenstein CP et al. The cohesive metastasis phenotype in human prostate cancer. *Biochim Biophys Acta* 2016;1866:221–31. 10.1016/j.bbcan.2016.09.005 PMC5534328 27678419

[41] Ulintz PJ, Greenson JK, Wu R et al. Lymph node metastases in colon cancer are polyclonal. *Clin Cancer Res* 2018;24:2214–24. 10.1158/1078-0432.CCR-17-1425 PMC5932232 29203589

[42] Liu X, Taftaf R, Kawaguchi M et al. Homophilic CD44 interactions mediate tumor cell aggregation and polyclonal metastasis in patient-derived breast cancer models. *Cancer Discov* 2019;9:96–113. 10.1158/2159-8290.CD-18-0065 PMC6328322 30361447

[43] Taftaf R, Liu X, Singh S et al. ICAM1 initiates CTC cluster formation and trans-endothelial migration in lung metastasis of breast cancer. *Nat Commun* 2021;12:4867. 10.1038/s41467-021-25189-z PMC8358026 34381029

[44] Aktipis A . The Cheating Cell: How Evolution Helps us Understand and Treat Cancer. Princeton, NJ: Princeton University Press, 2020.

[45] Hershey BJ, Barozzi S, Orsenigo F et al. Clonal cooperation through soluble metabolite exchange facilitates metastatic outgrowth by modulating Allee effect. *Sci Adv* 2023;9:eadh4184. 10.1126/sciadv.adh4184 PMC10881076 37713487

[46] Compton Z, Hanlon K, Compton CC, Aktipis A, Maley CC. A Missing Hallmark of Cancer: Dysregulation of Differentiation. arXiv [q-bio.PE]. 2022. Available: http://arxiv.org/abs/2210.13343

[47] Hanahan D . Hallmarks of cancer: New dimensions. *Cancer Discov* 2022;12:31–46. 10.1158/2159-8290.CD-21-1059 35022204

[48] Rofstad EK, Mathiesen B, Kindem K et al. Acidic extracellular pH promotes experimental metastasis of human melanoma cells in athymic nude mice. *Cancer Res* 2006;66:6699–707. 10.1158/0008-5472.CAN-06-0983 16818644

[49] Riemann A, Rauschner M, Gießelmann M et al. Extracellular acidosis modulates the expression of epithelial-mesenchymal transition (EMT) markers and adhesion of epithelial and tumor cells. *Neoplasia.* 2019;21:450–8. 10.1016/j.neo.2019.03.004 PMC6447725 30953950

[50] Lee S, Shanti A. Effect of exogenous pH on cell growth of breast cancer cells. *Int J Mol Sci* 2021;22:9910. 10.3390/ijms22189910 PMC8464873 34576073

